# Can Dairy Slurry Application to Stubble, without Incorporation into the Soil, Be Sustainable?

**DOI:** 10.3390/plants11111473

**Published:** 2022-05-31

**Authors:** Arejacy A. Silva, Mario Carvalho, João Coutinho, Ernesto Vasconcelos, David Fangueiro

**Affiliations:** 1 Instituto Federal de Educação, Ciência e Tecnologia de São Paulo, Avenida Professor Celso Ferreira da Silva 1333, Avare 18707-150, Brazil; 2 LEAF, Instituto Superior de Agronomia, Universidade de Lisboa, Tapada da Ajuda, 1349-017 Lisbon, Portugal; evasconcelos@isa.ulisboa.pt; 3 MED, Universidade de Evora, 7005-399 Evora, Portugal; mariogpcarvalho@gmail.com; 4 Centro de Química, Universidade de Trás-os-Montes e Alto Douro, 5000-911 Vila Real, Portugal; j_coutin@utad.pt

**Keywords:** acidified slurry, mineral fertilizer, nitrogen, nutrient use efficiency, no-tillage, conservation agriculture, ryegrass, ARN, N-MFE

## Abstract

In many countries, livestock slurry must be injected or incorporated into the soil to reduce nitrogen losses. However, when the injection is not feasible, farmers adopting conservation practices discard the use of slurry as fertilizer. New approaches related to slurry treatment or application management can stimulate the use of slurry in conservation agriculture (CA). This study aimed to evaluate the agronomic effects of some new management strategies to use dairy slurry for fertilization of ryegrass grown on stubble-covered soil, using as reference standard practices (slurry injection and mineral fertilizer application). The following treatments were considered: (i) bare soil: control (CB), mineral fertilizer (MB), injection (IN); (ii) stubble: control (CS), acidified dairy slurry (ADS), raw dairy slurry (RDS), irrigation following RDS (IR), mineral fertilizer (MS), RDS placed under the stubble (US), raw slurry applied 16 days after sowing (RDS T16). Effects on ryegrass yield, apparent nutrient recovery (ANR) and soil chemical properties were assessed. ADS reached 94% equivalence to MS and performed similarly to IN for productivity, ANR and soil parameters showing to be a sustainable alternative to replace mineral nitrogen and a potential solution to enable dairy slurry application in CA without injection or incorporation into the soil.

## 1. Introduction

The growing global demand for food, fibres and biofuels, in parallel with new priorities such as climate change and optimization of natural resources use, constitute a complex challenge: to increase crop yields while maintaining the focus on reducing pressures on the environment [1,2,3]. Therefore, new models of crop production must be based on solutions that preserve or increase soil health and productivity and enable the reuse of residues to stimulate nutrient recycling [4]. Additionally, the adoption of technologies able to increase crop resilience to climate change (e.g., more frequent droughts, high-intensity rainfall) is paramount.

Conservation agriculture (CA) practices (e.g., no-tillage, orchard row-middle sod maintenance) contribute to the sustainability of the agricultural system, helping to cope with and having the potential to increase land productivity [5,6,7,8,9,10]. By avoiding soil disturbance and by maintaining soil cover, CA has been promoted to increase the soil organic matter content, improve soil physical structure [5,7,11,12], maintain the soil moisture [7,8,9,10,13] and protect soil from erosion [12,14,15,16]. Preventing soil erosion is fundamental for southern Europe due to episodic heavy precipitation events typical of the autumn and winter seasons in Mediterranean-type climates [17,18].

Livestock slurry application to soil provides nutrients to crops and is also a source of organic matter and useful microorganisms, determining factors for the health and productivity of the soil [19,20]. However, due to the potential nitrogen (N) losses, mainly by ammonia emission, field slurry application has to be carried out using loss-mitigating practices, and slurry injection is the recommended technique in most countries of the European Union [17]. Nevertheless, because of the farmland characteristics or to avoid strong machinery investments, in countries from southern Europe as well as in South America, the slurry is mostly applied by broadcast, followed by incorporation into the soil [21,22,23] given that, if the slurry is left on the soil surface, a substantial nitrogen loss by ammonia emission could be expected [24].

As soil disturbance is undesirable in conservation crop systems, slurry incorporation into the soil should be avoided. Therefore, to promote the use of livestock slurry together with CA practices, it is necessary to develop and spread strategies that can achieve results similar to those obtained with the slurry injection or mineral/synthetic fertilizers application. Other strategies related to slurry fertilization, some of which have already been assessed in conventional crop systems, should be evaluated in stubble-covered soil in search of solutions that allow exemption from injection or incorporation into the soil. Slurry acidification is a well-studied technology, with the ability to reduce ammonia emissions, already in use on a farm scale [21,22]. The transport of the total ammoniacal nitrogen downward into the soil, by irrigation water, might also reduce the N lost from slurries applied to the soil surface [25]. Applying slurry only after plants’ emergence allows nutrients supply to plants when required, reducing the risk of N losses also, this strategy allows to extend the slurry application window, alleviating work overload of planting times [26]. Besides, if the slurry is placed under the stubble layer, protected from solar radiation and airflow, the ammonia emissions might be lessened and such a field-scale operation would require much less effort than injection. Therefore, we hypothesize that the potential of these strategies to reduce N losses in stubble-covered soil can lead them to reach levels of nutrient recovery and ryegrass productivity similar to those provided by the slurry injection and by mineral fertilizer.

The animal slurry application might be a solution to improve economically and environmentally the conservation agriculture system. However, studies on solutions able to obviate slurry injection and incorporation into the soil and minimise N losses are scarce. Thus, this study aimed to evaluate the agronomic effects of some new management strategies for dairy slurry fertilization in stubble-covered soil. These strategies were compared with raw dairy slurry and standard practices (slurry injection and mineral fertilizer) on the ryegrass productivity, nutrient recovery and soil physicochemical properties, expecting that our results can serve to update regulations for the use of manure in conservation agriculture systems, thus promoting the valorisation and proper use of livestock residues in agriculture.

## 2. Results

### 2.1. Ryegrass Aboveground Biomass

The total and partial (for each harvest) ryegrass DM yield, as well as the percentage contribution of each harvest of aboveground biomass to the total productivity, are presented in Table 1.

The time elapsed from the emergence of the plants to the first harvest was 59 days, and the interval from the beginning of regrowth to the next harvest was, respectively, 28, 25 and 83 days for the second, third and fourth harvests. The results showed an increasing trend in the ryegrass yield until the third harvest, more intense for the fertilized treatments than for Controls. The total DM yield of fertilized treatments was significantly greater than in CS and CB (Controls). ADS achieved the highest total DM yield, significantly greater (~40%) than RDS T16, but statistically similar to the other fertilized treatments. Regarding ryegrass productivity, fertilization with slurry or mineral fertilizer behaved similarly, as well as the application of fertilizers to stubble or bare soil (Table 1).

### 2.2. Nutrient Recovery

**Figure 1:**
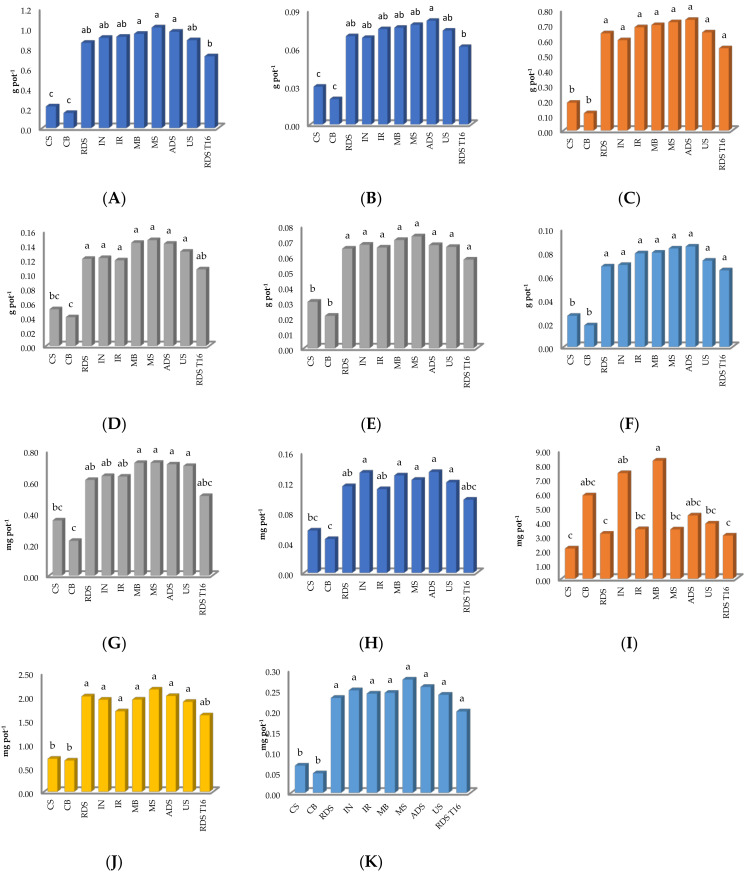
Nutrients recovered by aboveground ryegrass biomass harvesting (Means of three replicates). Nitrogen (**A**), Phosphorus (**B**), Potassium (**C**), Calcium (**D**), Magnesium (**E**), Sulphur (**F**), Zinc (**G**), Boron (**H**), Iron (**I**), Manganese (**J**), Copper (**K**). Different letters on the columns indicate significant differences at *p* < 0.05 for treatments, according to the Tukey test.

The nutrient export reflects the amount of nutrients recovered from the soil by harvested ryegrass aboveground biomass throughout the experiment, as an effect of each treatment. The recovery of the different nutrients presented a similar pattern among the treatments, as seen in Figure 1. Organic fertilizers performed comparably to mineral fertilizers when applied to stubbles or bare soil. The results were also similar between surface application without incorporation and slurry injection into the soil. Overall, fertilizers allowed greater nutrient recovery than controls, except for iron. ADS should be highlighted as it promoted the greater recovery of P despite being significantly greater only than RDS T16. Nitrogen recovery in ADS was greater than in RDS T16 and equivalent to MS and MB. Furthermore, RDS T16 presented the lowest value of nutrient recovery among the fertilized treatments, although in most cases, no significant differences were observed.

The nutrient export rates were also useful to assess the nutrient use efficiency of the treatments. The apparent nutrient recovery efficiency for N, P and K, is shown in Table 2. The highest N-ANR was reached by MS, MB, ADS and IN, with values significantly higher than RDS T16 but similar to IR, US and RDS. MB led to the highest value of P-ANR, but it was only significantly greater than RDS T16. There were no differences among treatments for K-ANR.

**Figure 2:**
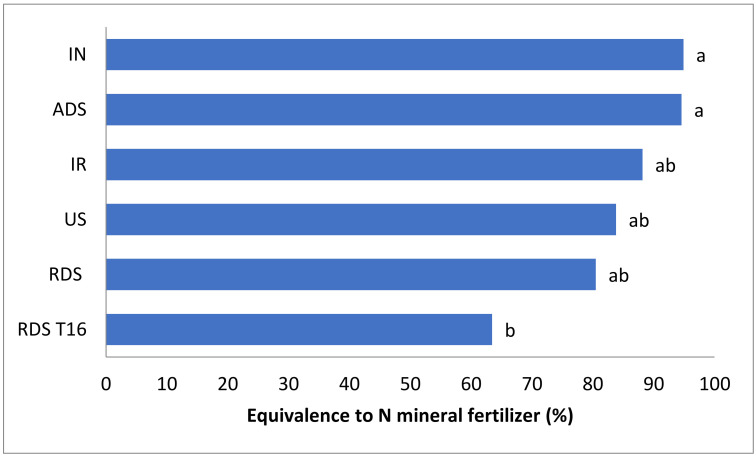
Nitrogen mineral fertilizer equivalence (Means of three replicates). Different letters on the columns indicate significant differences at *p* < 0.05 for treatments, according to the Tukey test.

Through the N-ANR of the treatments, it was possible to access the nitrogen mineral fertilizer equivalent (N-MFE) shown in Figure 2. The N-MFE may be used to describe how efficient certain manure, or management strategy of manure use, is in providing available nitrogen for the plants compared with a mineral/synthetic source of nitrogen [27]. Ammonium sulphate was the mineral fertilizer used in this study due to its efficiency in providing nitrogen to plants [28,29,30,31]. The higher the equivalence, the greater the ability of a given organic fertilizer (treatment) to replace mineral fertilizer, as a nitrogen source.

IN and ADS were the treatments that most efficiently (~94%) replaced ammonium sulphate as a nitrogen source for ryegrass production. On the other hand, RDS T16 had the lowest performance for the replacement capacity of ammonium sulphate, with close to 63% of efficiency. Lastly, IR, US and RDS equivalences to ammonium sulphate achieved, respectively 88%, 84% and 80%, although, without significant differences from IN and ADS.

### 2.3. Effects on the Soil

The effect of treatments on the soil’s chemical properties is presented in Table 3. Significant differences among treatments were observed only for soil pH and electrical conductivity (EC). There was a wide variation in pH among treatments, although, excluding CB and CS, no significant differences were observed among the treatments. Fertilizers affected the soil EC, with the fertilized treatments presenting values significantly greater than Controls.

## 3. Discussion

### 3.1. Ryegrass Yield

**Figure 3:**
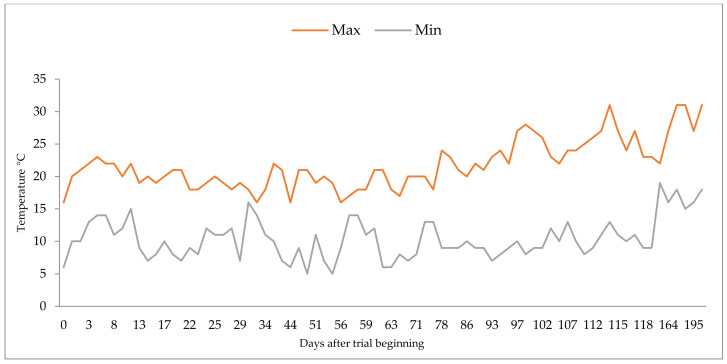
Daily temperatures in the agricultural greenhouse during the trial period.

A trend of increasing ryegrass aboveground biomass productivity, more intense in the fertilized treatments, was observed mainly from the first to the third harvest (Table 1). The low ryegrass yield at the beginning of the trial might have been an effect of the low temperatures (Figure 3) and weak luminosity, typical of late autumn and early winter in Portugal. These abiotic factors, as stressors, may have impaired nutrient and water uptake to some degree and thus affected plant growth. The optimum temperature for ryegrass production is around 18 to 20 °C and tillering is maximized at temperatures of 13 to 25 °C [32,33]. A slight increase in temperature after the first harvest had positive effects on the growth rate of the plants, accelerating plant development during the second and third ryegrass growing periods. On the other hand, after the third harvest, the reduced soil moisture and the absence of new fertilization reduced the growth rate and enlarge the time until the next harvest, as reported by [34,35].

The performance of the controls in the first harvest was possibly due to the high soil fertility but, on the other hand, this might also be an indication that some immobilization of nutrients occurred after fertilizers application, thus representing another stressor to impair the initial growth of plants in treatments that received fertilization. The 16-day delay in the slurry application led to a reduction in ryegrass productivity in the first harvest contributing to the lowest total yield of RDS T16 among the fertilized treatments, even if only significantly different from ADS. Such a decrease in production might be due to the absence of fertilization in the first 16 days followed by a possible period of nutrients immobilization, during a phase of increasing demand by the plants [36].

The better performance of ADS, MB and MS might be related to their influence on the slight reduction of soil pH (Table 3). The acidic soil pH, close to neutrality, tends to promote the greater availability of nutrients to plants [37]. Many plants prefer slightly acidic soil conditions, such as annual ryegrass, which requires a soil pH range between 5.3 and 7.0 [38]. Additionally, it is noticeable that the different slurry application strategies achieved ryegrass aboveground biomass yield similar to that of mineral fertilizer applied to stubble. Furthermore, the ryegrass yield promoted by MB and IN did not differ significantly from the dairy slurry treatments applied on the soils covered by wheat residues. In summary, regarding ryegrass yield, the strategies evaluated in this study showed similar behaviour to the dairy slurry injection into the soil and fertilization with ammonium sulphate. Even though the main goal of the present study was not to compare conventional tillage and no-tillage systems, it is noteworthy that the ryegrass yield obtained in CS was higher than in CB, likely as a result of stubble’s ability to hold soil moisture. Stubble-covered soils usually keep the moisture for longer than bare soils, favouring the absorption of water and nutrients [5,39].

### 3.2. Nutrients Use Efficiency

The recovery of nutrients by plants makes it possible to infer the availability of the nutrients in the soil, which influences crop production as well as the nutrient concentration in plants. The general pattern of nutrients exported through the harvests demonstrated that fertilized treatments led to greater nutrient recovery than Controls (Figure 1). ADS, IR, IN, US and RDS presented nutrient recovery rates similar to MS, MB and IN, for macronutrients and micronutrients, except for iron. Experimental data are not sufficient to explain exactly the causes of variation in the iron export rates among the treatments. Interestingly, the treatments deployed on bare soil presented higher levels of exported iron than those on stubble-covered soil. The lower iron uptake by plants can be related to several factors, for instance, the interactions with P, Zn, Mn, Cu, neutral or alkaline soil pH, high soil moisture and low temperatures [40,41]. Thus, as less temperature variation at the soil surface occurred in stubble-covered soils [42], it is possible that, especially on colder days, the night-time temperatures of the soil surface covered by stubbles remained below than in bare soil, leading to a lower iron uptake by plants. However, it is important to note that there were no symptoms of iron deficiency or toxicity in plants of any pot. RDS T16 presented lower phosphorus export than ADS and lower nitrogen export than ADS, MS and MB. In addition, ADS stood out in the amount of nitrogen and phosphorus exported through aboveground biomass harvest. Higher levels of N and P in plants contribute to an increase in the protein content and nutritional value of food for humans and animals [43,44].

Among the strategies evaluated, ADS was the one that presented the ANR values closest to MB, MS and IN, thus showing its potential to replace them when applied to stubble-covered soils, without incorporation into the soil. Greater nutrient use efficiency is commonly related to the greater nutrient availability in the soil and at the same time, to the lower nutrient losses such as gas emissions and leachate [45,46]. MS, MB, ADS and IN led to the highest values of N-ANR, significantly superior to RDS T16. Several studies have confirmed the ability of acidified slurry and ammonium sulphate to reduce ammonia emissions [30,47,48,49] while decreasing soil pH [30,50,51,52]. Additionally, a soil pH slight below the neutrality can improve the nutrient availability for plants [37,53]. As raw slurries are highly prone to ammonia emission after field application [54,55], a significant nitrogen loss by ammonia emission could help to explain the low N-ANR of RDS and RDS T16. Moreover, RDS T16 may have been affected also by a possible nutrient deficiency in the early days, as previously reported. MB achieved the highest P-ANR with no differences from the others, except for RDS T16. Despite the small numerical difference from MB, the P-ARN of ADS was below expectation. The acidification ability to dissolve the slurry inorganic P could lead to higher P recovery and P-ANR, probably the P uptake was impaired as an effect of the soil buffer capacity as well as the high *p* sorption capacity [56]. In addition, the soil sorption capacity and the relatively low P requirement of ryegrass [57] appear to be the main reasons why P-ANR was lower than N-ANR [46]. The apparent K recovery efficiency was similar among the treatments, likely because the K input was levelled among the treatments.

### 3.3. Nitrogen-Mineral Fertilizer Equivalence

The nitrogen mineral fertilizer equivalence (N-MFE) is an efficiency indicator that describes how efficient an organic fertilizer is in providing available nitrogen for the plants compared with a mineral fertilizer during the first year after application. Factors related to manure such as manure type, the application method and application timing can affect the N-MFE and consequently, there is no universal value for a particular manure type [27]. Moreover, N-MFE may vary depending on the soil texture [57] and the mineral fertilizer used as reference. The greatest equivalences to ammonium sulphate were achieved by IN and ADS, (~94%) significantly greater than RDS T16, despite they were similar to RDS, IR and US (Figure 2). Indeed, according to [57], slurry application methods that efficiently avoid NH_3_ losses, such as IN and ADS, may increase the N-MFE. N-MFE values from 39% to 88%, for cattle slurry in field application, have been reported by [57]. The N-MFE obtained in the present study, with emphasis on ADS and IN, was possibly due to the well-controlled conditions that contributed to reducing N losses, such as leaching.

### 3.4. Effects on Soil Properties

There were significant variations in soil pH between controls and fertilized treatments (Table 3). MB and MS were the treatments that most acidified the soil (0.8 and 0.46 units compared to CB and CS, respectively), although without significant differences relative to the other fertilized treatments. The acidic reaction of ammonium sulphate in the soil may explain the acidification effect caused by MB and MS on the soil [58]. The high soil buffer capacity inferred given its high CEC (Table 4), possibly limited a more intense decrease in soil pH. Therefore, the slurry acidity in ADS reduced the soil pH, at a similar level to MB and MS and even though it was not significantly different from the other treatments, it might have been enough to promote higher nutrient availability to the plants [37], positively impacting the ryegrass yield. On the other hand, long-term fertilizer applications that cause an acidic reaction in the soil, especially in low CEC soils, can lead to a decline in nutrient availability [59]. The soil electrical conductivity (EC) was influenced by the fertilizers applied to soils, since soil EC was significantly higher for amended treatments than for controls, as an effect of the nutrients and salts added through organic and mineral fertilizers and the influence of these fertilizers on solubilisation, sorption, mineralization and immobilization rates in the soil [60,61]. Dairy slurry, regardless of the method adopted in the application, affected the soil EC at a similar level to ammonium sulphate.

In contrast to the mineral fertilizers, dairy slurry supplies simultaneously several nutrients (Table 5) that can contribute to the increased crop yield. However, unlike what is reported in long-term experiments [46,59], our results showed that there were no significant differences in soil P, K, Ca, Mg and Na levels between mineral fertilizer and dairy slurry-based treatments. As P and K inputs were levelled among the treatments, the absence of differences was already expected. For the others, the reason is likely associated with the soil pH and also its high cation exchange capacity. Despite the carbon provided by the dairy slurry and the ability of no-tillage to increase soil organic matter [62,63,64,65], changes in soil organic matter content require a longer time [66,67].

## 4. Materials and Methods

### 4.1. Soil, Slurry and Wheat Stubble

A Vertic Cambisol from an area usually cultivated with wheat at Instituto Superior de Agronomia in Lisbon, Portugal (coordinates 38°42′29.786″ N; 9°11′6.18″ W) was collected to fill the experimental pots. An undisturbed 5 cm top layer soil monolith and disturbed soil from the complementary 5–20 cm layer were collected. The sampled soil was air-dried at ambient temperature for eight weeks before beginning the study.

The physical characteristics of the soil (0–20 cm) used were: texture (16.8% coarse sand, 33.7% fine sand, 20.9% silt and 28.6% clay), water holding capacity (WHC): 348.8 g kg^−1^. The main chemical characteristics of the two layers are described in Table 4.

The dairy slurry was collected from the storage tank of a typical commercial dairy farm located in the Setubal region, Portugal, and stored at ambient temperature in plastic barrels loosely covered, until further treatment and utilization. Before use or sampling, the slurry was stirred manually until a homogeneous material was obtained.

In the treatments where no-tillage agriculture was simulated, the soil surface in each pot was covered with the equivalent of 300 g m^−2^ of wheat stubble collected in the same area of the soil. The main characteristics of dairy slurry and wheat stubble are presented in Table 5.

### 4.2. Experimental Design

The pot experiment was carried out for six months in a greenhouse, to evaluate the impact of the dairy slurry management strategies applied to stubble-covered soil. The parameters measured were ryegrass aboveground biomass, nutrient uptake, nitrogen, phosphorus and potassium use efficiency, and soil status compared to the standard practices and a non-fertilized soil. Daily temperatures inside the greenhouse during the experimental period are shown in Figure 3.

Polyvinyl chloride pots (Ø = 250 mm, h = 200 mm; capacity = 6000 cm^3^) were filled with 7 kg of dry soil. A 15 cm layer of disturbed soil was placed at the bottom of the pots. A 5 cm undisturbed monolith was then placed on the top of that layer.

To be able to compare the results obtained in no-tillage conditions with conventional practices, three treatments were installed in bare soil, as reference: slurry injection, surface application of mineral fertilizer and a control. All treatments, except RDS T16, were applied on the day of sowing.

Thus, the treatments considered here were separated into two groups: (i) bare soil, and (ii) stubble-covered soil.

i. unfertilized bare soil, control (CB), injected slurry in bare soil (IN), mineral fertilizer applied on bare soil (MB);
ii. unfertilized stubble-covered soil, control (CS), raw dairy slurry on the stubble (RDS), acidified dairy slurry on the stubble (ADS), irrigation just after RDS application (IR), mineral nitrogen on the stubble (MS), raw slurry applied under the stubble (US), 16-day delayed application of RDS (RDS T16).

A completely randomized experiment was performed with 3 replications, resulting in a total of 30 pots. The pots were placed on a table that allowed to rotate the pot positions, so the positions were changed daily.

Annual ryegrass (*Lolium multiflorum*) sowing (16 seeds in two parallel rows) was carried out on the same day in all pots, and as soon as possible, the surplus seedlings were uprooted thus making the final population of 10 plants per pot. The plants emerged from the soil seven days after the sowing. The basal fertilization for all treatments, except for RDS T16, was applied just after the sowing.

For IN treatment, the slurry was placed at 5 cm depth into two channels parallel to seed lines. The source of mineral nitrogen (MB and MS) was ammonium sulphate (20.5% of total nitrogen). For IR treatment, the rate of irrigation applied on the slurry was equivalent to 10 mm, and this amount of water was considered when soil moisture was corrected. In US treatment, the slurry was placed on the soil surface and then covered with wheat stubble. The US is a conceptualization based on the protection provided by stubble against abiotic factors (i.e., wind and solar radiation) on slurry possibly leading to lower N loss by ammonia emissions and higher nutrient recovery efficiency, as reported by [68]. For the slurry acidification in ADS, concentrated sulphuric acid was added to the raw slurry until it reached a pH = 5.5, monitored by a pH meter. Lastly, for the RDS T16 treatment, the slurry was applied only at the ryegrass tillering stage, 9 days after the emergence of the plants (16 days after sowing). The soil moisture in the pots until the third harvest was kept close to 70% of the soil water holding capacity (WHC), by regular addition of deionised water. From the third harvest to the end, the WHC was kept close to 50%, simulating the spring season weather condition in Central Portugal. No leachate was produced, and consequently, no nutrients were lost via leaching.

Due to the soil physicochemical characteristics and the low phosphorus (P) content in the slurry, the input of P_2_O_5_ was 0.22 g P per pot for all treatments, except for CS and CB, through the application of 2.75 g of simple superphosphate for the treatments without slurry or 1.1 g for those amended with slurry. Additionally, the treatments that were not fertilized with slurry, received 0.66 g K per pot, through the application of 1.3 g potassium chloride, equivalent to the amount delivered by the dairy slurry.

The total-nitrogen supply was 1.5 g N per pot, divided into three applications: 0.5 g (155 g dairy slurry or 2.4 g ammonium sulphate, depending on the treatment) right after the sowing (16 days later for RDS T16), plus 0.5 g (2.4 g ammonium sulphate for all treatments, except CS and CB) by top dressing after each of the two first harvests, as is usually done in commercial crops. The difficulties and uncertainties caused by the first wave of the COVID-19 pandemic led us not to perform new fertilization after the third harvest.

Four harvests of whole biomass above 10 mm from the soil surface were made manually at 66, 94, 119 and 202 days after sowing.

### 4.3. Analytical Methods

For the first ryegrass aboveground biomass harvest, due to the low dry matter (DM) yield of some treatments, nitrogen was the only nutrient analysed, then a complete analysis of ryegrass was carried out from the second to the fourth harvests. The ryegrass and wheat stubble DM were determined after oven-drying at 65 °C to constant weight. Dried ryegrass and wheat stubble samples were ground at 1 mm in a grain mill. Wheat stubble analysis procedures are described in [49]. For the ryegrass, the total-nitrogen was determined by the Kjeldahl method, following the digestion, distillation, and titration steps [69], while the NH_4_^+^ − N content was determined directly by distillation and titration. Other nutrients (P, K, Ca, Mg, S, B, Cu, Fe, Mn, Na, Zn) were determined by acid digestion at 105 °C with a 9:3 (*v*/*v*) mixture of HCl (37% (*w*/*w*) HCl, d = 1.19 kg L^−1^) and HNO_3_ (65% (*w*/*w*) HNO_3_, d = 1.39 kg L^−1^), respectively, followed by quantification by inductively coupled plasma—mass spectrometry (ICP-EOS).

Slurry analyses were performed as described by [49]. The soil pH and EC were determined in a soil: water (1:2.5 *w*/*v*) suspension. The methods used to determine soil texture, organic matter, total and mineral N, extractable P and K are fully described in [49]. Cation exchangeable and extractable micronutrients were performed as described by [70].

### 4.4. Nutrient Use Efficiency-Related Indicators

The apparent nutrient recovery efficiency was the agronomic indicator used to evaluate the nutrient use efficiency by plants, as it represents the plant’s ability to acquire applied nutrients [45].

For each fertilized treatment, the apparent nutrient recovery efficiency, expressed in g g^−1^, was calculated as presented in Equation (1):

(1) $$Y-\mathrm{ANR}=\frac{\left(\mathrm{Exported}\;Y\;\mathrm{treatment}-\mathrm{Exported}\;Y\;\mathrm{control}\right)}{\mathrm{Applied}\;Y}\;.\;$$

where Exported Y _treatment_ is the Y (N, P or K) recovered by ryegrass aboveground biomass from a given evaluated fertilized treatment, and Exported Y _Control_ is the Y recovered by ryegrass aboveground biomass in control treatments (CB or CS). Applied Y is the amount of nutrient (N, P or K) applied per pot [39].

According to [27], the ability of a treatment, based on organic fertilizer, to replace the mineral fertilizer as an available nitrogen source, is calculated through the nitrogen mineral fertilizer equivalent (N-MFE), Equation (2).

(2) $$N-\mathrm{MFE}=\frac{N-\mathrm{ANR}\;\mathrm{treatment}}{N-\mathrm{ANR}\;\mathrm{MF}}$$

where N-ANR treatment is the Apparent N recovery efficiency of a given treatment based on organic fertilizer, and ANR MF is the Apparent N Recovery efficiency of the mineral fertilizer, in this case, ammonium sulphate (MB or MS).

### 4.5. Statistical Analysis

All results were analyzed using the Analytical Software Statistix 9 program (Analytical Software, Tallahassee, FL, USA) The effects of the treatments were tested by analysis of variance (one-way ANOVA). Differences were considered statistically significant at a *p* < 0.05 level of probability. Tukey’s test was used to analyse the differences in the mean values of the parameters between treatments. Log-transformations of variables were performed when necessary to ensure normality and homogeneity of the variances.

## 5. Conclusions

The dairy slurry application to stubble-covered soil as basal fertilization influenced ryegrass aboveground biomass yield and ANR (N, P and K) similarly to IN, MB and MS. Besides, the slurry N-MFE was also similar between IN and the treatments applied to stubble without incorporation into the soil. Due to poor performance, RDS T16 proved not to be a viable solution. However, shorter periods of delay in fertilization should be evaluated.

The effects of treatments on soil properties and other response variables influenced by these properties were probably affected by high soil fertility. Therefore, new studies on coarse-textured, low-fertility soil will contribute to a wider comprehension of the agronomic effects of the presented strategies in different soil conditions.

We can conclude that the treatments ADS, IR and US considered here are sustainable strategies for application to stubble-covered soil without incorporation into the soil, having the potential to reach levels of nutrient recovery efficiency and ryegrass productivity similar to those provided by the slurry injection and by mineral fertilizer.

Among the strategies evaluated, ADS can be highlighted due to its good and consistent performance. This strategy presented the greatest potential to enable the application of dairy slurry in CA without the need for injection or incorporation into the soil. This work can contribute to updating regulations promoting and stimulating the proper use of livestock residues in conservation agriculture systems.

The evaluation of the solutions presented in this work on a field scale is important to corroborate the results obtained in pots. Further studies addressing the effects of ADS, IR and US on ammonia and nitrous oxide emissions are needed to enable a broader understanding of the solutions presented in this work.

## Figures and Tables

**Table 1:** Ryegrass aboveground DM yield and as a percentage of total yield (%) obtained at each harvest. Values presented are arithmetic means of three replicates. For each parameter, values followed by different letters in the same column, are significantly different based on the Tukey test (*p* < 0.05).

Treatment	Harvest								Total Yield
	1		2		3		4		
	DM g Pot^−1^	%	DM g Pot^−1^	%	DM g Pot^−1^	%	DM g Pot^−1^	%	DM g Pot^−1^
CB	0.92 ^ab^	16.77	1.10 ^c^	20.18	1.30 ^d^	23.84	2.14 ^c^	39.21	5.47 ^d^
CS	1.04 ^ab^	12.64	1.87 ^bc^	22.72	1.51 ^d^	18.31	3.81 ^bc^	46.33	8.23 ^c^
IN	0.92 ^ab^	4.69	2.63 ^ab^	13.37	9.55 ^ab^	48.52	6.58 ^ab^	33.42	19.69 ^ab^
MB	0.92 ^ab^	4,22	3.03 ^ab^	13,90	9.15 ^abc^	41.93	8.72 ^a^	39.95	21.82 ^ab^
MS	1.30 ^a^	5.54	3.67 ^a^	15.67	9.48 ^ab^	40.49	8.97 ^a^	38.30	23.42 ^ab^
RDS	0.91 ^ab^	4.60	1.95 ^bc^	9.82	8.65 ^abc^	43.53	8.35 ^a^	42.05	19.86 ^ab^
ADS	1.21 ^ab^	4.94	3.19 ^ab^	13.01	10.65 ^a^	43.45	9.46 ^a^	38.60	24.52 ^a^
IR	0.83 ^ab^	3.80	2.52 ^ab^	11.61	9.40 ^ab^	43.26	8.98 ^a^	41.33	21.74 ^ab^
US	1.15 ^ab^	5.52	3.04 ^ab^	14.61	7.69 ^bc^	36.91	8.95 ^a^	42.96	20.83 ^ab^
RDS T16	0.69 ^b^	3.96	1.92 ^bc^	11.05	6.41 ^c^	36.83	8.38 ^a^	48.15	17.40 ^b^

Treatments: Unfertilized bare soil (CB), unfertilized stubble-covered soil (CS), Injected slurry into bare soil (IN), Mineral fertilizer applied on bare soil (MB), Mineral fertilizer on the stubble (MS), Raw slurry on the stubble (RDS), Acidified Slurry on the stubble (ADS), Irrigation just after raw slurry applied on the stubble (IR), Raw slurry applied under the stubble (US), 16-day raw slurry delayed application on the stubble (RDS T16). Different lowercase letters within the same column indicate significant difference at *p* < 0.05.

**Table 2:** N, P and K apparent nutrient recovery efficiency (ANR) of evaluated treatments, means of three replicates. For each parameter, values followed by different letters in the same column, are significantly different based on the Tukey test (*p* < 0.05). The absence of letters in the column indicates non-significant differences.

Treatment	N (g g^−1^ Applied N)	P (g g^−1^ Applied P)	K (g g^−1^ Applied K)
MB	0.53 ^a^	0.26 ^a^	0.90
MS	0.53 ^a^	0.22 ^ab^	0.82
ADS	0.50 ^a^	0.24 ^ab^	0.84
IN	0.50 ^a^	0.22 ^ab^	0.74
IR	0.46 ^ab^	0.21 ^ab^	0.77
US	0.44 ^ab^	0.20 ^ab^	0.72
RDS	0.42 ^ab^	0.18 ^ab^	0.71
RDS T16	0.33 ^b^	0.15 ^b^	0.55

Treatments: Mineral fertilizer applied on bare soil (MB), Mineral fertilizer on the stubble (MS), Acidified Slurry on the stubble (ADS), Injected slurry into bare soil (IN), Irrigation just after raw slurry applied on the stubble (IR), Raw slurry applied under the stubble (US), Raw slurry on the stubble (RDS), 16-day raw slurry delayed application on the stubble (RDS T16). Different lowercase letters within the same column indicate significant difference at *p* < 0.05.

**Table 3:** Final soil properties, mean of three replicates. For each parameter, values followed by different letters in the same column, are significantly different based on the Tukey test (*p* < 0.05). The absence of letters on the column indicates non-significant differences.

Treatments	pH	EC	OM	P	K	Ca^++^	Mg^++^	K^+^	Na^+^
	H_2_O	μS cm^−1^	g Kg^−1^	mg Kg^−1^		cmolc Kg^−1^			
CB	7.44 ^a^	142.5 ^c^	25.8	153.41	193.75	34.54	10.09	0.24	0.47
CS	7.28 ^ab^	158.1 ^bc^	28.5	158.47	211.67	32.96	9.63	0.25	0.52
IN	7.02 ^abc^	552.3 ^ab^	23.8	155.78	172.90	35.82	10.34	0.24	0.64
MB	6.64 ^c^	758.3 ^a^	28.3	148.71	192.64	34.61	10.73	0.27	0.66
MS	6.82 ^bc^	842.3 ^a^	27.5	136.21	183.58	33.94	10.40	0.28	0.53
RDS	7.05 ^abc^	618.3 ^a^	27.6	154.40	208.86	34.46	9.93	0.26	0.58
ADS	6.86 ^bc^	588.3 ^a^	32.3	142.66	182.28	34.96	10.71	0.25	0.67
IR	6.84 ^bc^	603.6 ^a^	28.3	156.95	188.61	34.35	10.62	0.26	0.70
US	7.11 ^abc^	611.6 ^a^	25.4	151.99	207.54	34.19	10.46	0.26	0.54
RDS T16	7.01 ^abc^	543.8 ^ab^	28.2	142.49	182.07	33.81	10.48	0.25	0.63

Treatments: Unfertilized bare soil (CB), unfertilized stubble-covered soil (CS), Injected slurry into bare soil (IN), Mineral fertilizer applied on bare soil (MB), Mineral fertilizer on the stubble (MS), Raw slurry on the stubble (RDS), Acidified Slurry on the stubble (ADS), Irrigation just after raw slurry applied on the stubble (IR), Raw slurry applied under the stubble (US), 16-day raw slurry delayed application on the stubble (RDS T16). Different lowercase letters within the same column indicate significant difference at *p* < 0.05.

**Table 4:** Main characteristics of the soil used (Mean, *n* = 3).

Parameters	0–5 cm Layer	5–20 cm Layer
Organic Matter (g kg^−1^)	35.4	34.5
pH (H_2_O)	7.08	7.13
EC (μS cm^−1^)	281.65	264.40
Extractable P (mg kg^−1^)	214.24	238.77
Mg (cmolc kg^−1^)	14.70	11.30
K (cmolc kg^−1^)	1.07	0.64
Ca (cmolc kg^−1^)	44.7	63.85
Na (cmolc kg^−1^)	1.19	0.35
Cu (mg kg^−1^)	0.81	1.15
Zn (mg kg^−1^)	0.36	0.36
Fe (mg kg^−1^)	452.87	450.50
Mn (mg kg^−1^)	889.33	882.73
CEC (cmolc kg^−1^)	61.94	77.08
Total-N (g kg^−1^)	1.67	1.76
Organic N (g kg^−1^)	1.65	1.74
NH_4_-N (mg kg^−1^)	8.72	9.60
NO_3_-N (mg kg^−1^)	12.07	10.73

**Table 5:** Main characteristics of the dairy slurry and wheat stubble (Mean, *n* = 3).

Parameters	Dairy Slurry	Wheat Stubble
Dry matter (g kg^−1^)	113.0	921.6
pH	7.5	-
EC (mS cm^−1^)	16.5	-
Total Organic C (g kg^−1^) *	304.6	416.4
Total N (g kg^−1^)	3.2	5.2
NH_4_-N (g kg^−1^)	1.2	-
Total P (g kg^−1^)	0.9	0.1
Total K (g kg^−1^)	4.2	5.4

* Based on dry matter.

## Data Availability

Not applicable.

## References

[1] EC (2019). Food, Feed, Fibres, Fuels. Enough Biomass for a Sustainable Bieconomy? The European Comission’s Science and Knowledge Service.

[2] FAO (2009). Global Agriculture Towards 2050. High Level Experts Forum—How to Feed the World in 2050.

[3] Westcott P., Trostle R. (2012). Long-Term Prospects for Agriculture Reflect Growing Demand for Food, Fiber and Fuel.

[4] Liniger H.P., Studer R.M., Hauert C., Gurtner M. (2011). Gurtner. Sustainable Land Management in Practice—Guidelines and best Practices for Sub-Saharan Africa.

[5] Carvalho M.D.C.S., Lourenço E. (2014). Conservation Agriculture—A Portuguese Case Study. J. Agron. Crop Sci..

[6] Crassweller R. (2017). Orchard Establishment—Row Middle and Tree Row.

[7] Kassam A., Friedrich T., Derpsch R. (2019). Global spread of Conservation Agriculture. Int. J. Environ. Stud..

[8] Michler J.D., Baylis K., Arends-Kuenning M., Mazvimavi K. (2019). Conservation agriculture and climate resilience. J. Environ. Econ. Manag..

[9] Pittelkow C.M., Liang X., Linquist B.A., Van Groenigen K.J., Lee J., Lundy M.E., Van Gestel N., Six J., Venterea R.T., Van Kessel C. (2015). Productivity limits and potentials of the principles of conservation agriculture. Nature.

[10] Steward P.R., Dougill A.J., Thierfelder C., Pittelkow C., Stringer L.C., Kudzala M., Shackelford G.E. (2018). The adaptive capacity of maize-based conservation agriculture systems to climate stress in tropical and subtropical environments: A meta-regression of yields. Agric. Ecosyst. Environ..

[11] Barão L., Alaoui A., Ferreira C., Basch G., Schwilch G., Geissen V., Sukkel W., Lemesle J., Garcia-Orenes F., Morugán-Coronado A. (2019). Assessment of promising agricultural management practices. Sci. Total Environ..

[12] Corsi S. (2019). Conservation Agriculture: Training Guide for Extension Agents and Farmers in Eastern Europe and Central Asia.

[13] Vastola A., Zdruli P., D’Amico M., Pappalardo G., Viccaro M., Di Napoli F., Cozzi M., Romano S. (2017). A comparative multidimensional evaluation of conservation agriculture systems: A case study from a Mediterranean area of Southern Italy. Land Use Policy.

[14] Phillips R.E., Thomas G.W., Blevins R.L., Frye W.W., Phillips S.H. (1980). No-Tillage Agriculture. Science.

[15] FAO (2018). Nutrient Flows an Environmental Impacts in Livestock Supply Chain: Guidelines for Assessment.

[16] Soane B.B., Ball B.C., Arvidsson J., Basch G.L., Moreno F., Roger-Estrade J. (2012). No-till in northern, western and south western Europe: A review of problems and opportunities for crop production and the environment to cite this version: HAL Id: Hal-00956463. Soil Tillage Res..

[17] Cameira M.D.R., Valente F., Li R., Surgy S., Abreu F.G., Coutinho J., Fangueiro D. (2019). Band application of acidified slurry as an alternative to slurry injection in Mediterranean winter conditions: Impact on nitrate leaching. Soil Tillage Res..

[18] EUROSTAT (2020). Agri-Environmental Indicator—Soil Erosion. https://ec.europa.eu/eurostat/statistics-explained/index.php?title=Agri-environmental_indicator_-_soil_erosion#Introduction.

[19] Kumar R.R., Park B.J., Cho J.Y. (2013). Application and environmental risks of livestock manure. J. Korean Soc. Appl. Biol. Chem..

[20] Ozlu E., Sandhu S.S., Kumar S., Arriaga F.J. (2019). Soil health indicators impacted by long-term cattle manure and inorganic fertilizer application in a corn-soybean rotation of South Dakota. Sci. Rep..

[21] Fangueiro D., Pereira J.L.D.S., Bichana A., Surgy S., Cabral F., Coutinho J. (2015). Effects of cattle-slurry treatment by acidification and separation on nitrogen dynamics and global warming potential after surface application to an acidic soil. J. Environ. Manag..

[22] Fangueiro D., Pereira J.L.D.S., Macedo S., Trindade H., Vasconcelos E., Coutinho J. (2017). Surface application of acidified cattle slurry compared to slurry injection: Impact on NH3, N2O, CO2 and CH4 emissions and crop uptake. Geoderma.

[23] Morino C.C. (2021). A Aplicação de Dejetos de Suínos no Solo Como Insumo. Escola Superior da CETESB. https://cetesb.sp.gov.br/escolasuperior/wp-content/uploads/sites/30/2021/08/Camila-Canesi-Morino_TCC-T2-2021-versao-final.pdf.

[24] UNECE (2014). Guidance Document on Preventing and Abating Ammonia Emissions from Agricultural Sources.

[25] Sommer S., Génermont S., Cellier P., Hutchings N., Olesen J.E., Morvan T. (2003). Processes controlling ammonia emission from livestock slurry in the field. Eur. J. Agron..

[26] Koelsch R. (2020). Extending the Manure Application Window: Post Plant Experiences. https://water.unl.edu/article/animal-manure-management/extending-manure-application-window-post-plant-experiences.

[27] Jensen L.S., Sommer S.G., Christensen M.L., Schmidt T., Jensen L.S. (2013). Animal Manure Fertilizer Value, Crop Utilisation and Soil Quality Impacts. Animal Manure: Recycling, Treatment and Management.

[28] Bittman S., Dedina M., Howard C.M., Oenema O., Sutton M.A. (2014). Options for Ammonia Mitigation: Guidance from the UNECE Task Force on Reactive Nitrogen.

[29] Bell M. (2015). A Review of Nitrogen use Efficiency in Sugarcane. Research Report of Sugarcane.

[30] Chien S.H., Gearhart M.M., Villagarcía S. (2011). Comparison of ammonium sulfate with other nitrogen and sulfur fertilizers in increasing crop production and minimizing environmental impact: A review. Soil Sci..

[31] Vieira R.F. (2017). Ciclo do Nitrogênio em Sistemas Agrícolas.

[32] Donaghy D., Fulkerson W. (2001). Principles for Developing an Effective Grazing Management System for Ryegrass-Based Pastures.

[33] Hunt W.F., Easton H.S. (1989). Fifty years of ryegrass research in New Zealand. Proc. N. Z. Grassl. Assoc..

[34] Ayanz A.S.M. (2008). Gramíneas de Interés Para Implantación de Praderas y la Revegetación de Zonas Degradadas.

[35] Hart J., Mellybye M.E., Young W.C., Silberstein T.B. (2011). Annual Ryegrass Grown for Seed (Western Oregon). Nutrient Management Guide.

[36] Dominico C.D.F.T., Lustosa S.B.C., De Ávila F.W. (2020). Acúmulo de matéria seca e absorção de nitrogênio, fósforo e potássio por azevém (Lolium multiflorum Lam.) cultivar BARjumbo. Res. Soc. Dev..

[37] Miller J.O. (2016). pH Affects Nutrient Availability.

[38] AWI, MLA (2008). Healthy Soils. Making More from Sheep.

[39] Plaza-Bonilla D., Cantero-Martínez C., Bareche J., Arrúe J.L., Lampurlanés J., Álvaro-Fuentes J. (2017). Do no-till and pig slurry application improve barley yield and water and nitrogen use efficiencies in rainfed Mediterranean conditions?. Field Crop. Res..

[40] Abreu C.A., Lopes A.S., Santos G.C.G., Novais R.F., Alvarez V.V.H., Barros N.F., Fontes R.L., Cantarutti R.B., Neves J.C.L. (2007). Micronutrientes. Fertilidade do Solo.

[41] IPNI (2009). Ferro. Nutri-Fatos: Informação Agronômica Sobre Nutrientes Para as Plantas.

[42] Kovaleski S., Heldwein A.B., Dalmago A.G., Cunha G.R., Fochesatto E., Gouvêa J.A., Liska B. Temperatura e Fluxo de Calor no Solo em Dossel de Canola em Função da Distribuição da Palha na Superfície, em Noites de Ocorrência de Geada. Proceedings of the XIX Congresso Brasileiro de Agrometeorologia.

[43] Imran, Amanullah (2021). Phosphorus and Boron Application Optimizing Biofortification of P and Productivity of French Bean (*Phaseolus vulgaris* L.). Commun. in Soil Sci. Plant Anal..

[44] The S.V., Snyder R., Tegeder M. (2021). Targeting Nitrogen Metabolism and Transport Processes to Improve Plant Nitrogen Use Efficiency. Front. Plant Sci..

[45] Dobermann A. Nutrient use efficiency–measurement and management. Proceedings of the IFA International Workshop on Fertilizer Best Management Practices.

[46] Ferreira P.A.A., Ceretta C.A., Lourenzi C.R., De Conti L., Marchezan C., Girotto E., Tiecher T.L., Palermo N.M., Parent L., Brunetto G. (2022). Long-Term Effects of Animal Manures on Nutrient Recovery and Soil Quality in Acid Typic Hapludalf under No-Till Conditions. Agronomy.

[47] Fangueiro D., Surgy S., Fraga I., Monteiro F., Cabral F., Coutinho J. (2016). Acidification of animal slurry affects the nitrogen dynamics after soil application. Geoderma.

[48] Forrestal P.J., Harty M., Carolan R., Lanigan G.J., Watson C.J., Laughlin R.J., McNeill G., Chambers B.J., Richards K. (2016). Ammonia emissions from urea, stabilized urea and calcium ammonium nitrate: Insights into loss abatement in temperate grassland. Soil Use Manag..

[49] Silva A.A., Fangueiro D., Carvalho M. (2022). Slurry Acidification as a Solution to Minimize Ammonia Emissions from the Combined Application of Animal Manure and Synthetic Fertilizer in No-Tillage. Agronomy.

[50] Buck G.B., De Castro G.F., Mattiello E.M., Zotarelli L. (2020). Applications of Gypsum and Ammonium Sulfate Change Soil Chemical Properties of a Salt-Affected Agricultural Soil. J. Agric. Sci..

[51] Fageria N.K., Dos Santos A.B., Moraes M.F. (2010). Influence of Urea and Ammonium Sulfate on Soil Acidity Indices in Lowland Rice Production. Commun. Soil Sci. Plant Anal..

[52] Pedersen I.F., Rubæk G.H., Sørensen P. (2017). Cattle slurry acidification and application method can improve initial phosphorus availability for maize. Plant Soil.

[53] Roboredo M., Fangueiro D., Lage S., Coutinho J. (2012). Phosphorus dynamics in soils amended with acidified pig slurry and derived solid fraction. Geoderma.

[54] Feilberg A., Sommer S.G., Sommer S.G., Christensen M.L., Schmidt T., Jensen L.S. (2013). Ammonia and Malodorous Gases: Sources and Abatement Technologies. Animal Manure Recycling: Treatment and Management.

[55] Sigurdarson J.J., Svane S., Karring H. (2018). The molecular processes of urea hydrolysis in relation to ammonia emissions from agriculture. Rev. Environ. Sci. Bio/Technol..

[56] Seidel A., Pacholski A., Nyord T., Vestergaard A., Pahlmann I., Herrmann A., Kage H. (2017). Effects of acidification and injection of pasture applied cattle slurry on ammonia losses, N_2_O emissions and crop N uptake. Agric. Ecosyst. Environ..

[57] Pantelopoulos A., Magid J., Jensen L.S., Fangueiro D. (2017). Nutrient uptake efficiency in ryegrass fertilized with dried digestate solids as affected by acidification and drying temperature. Plant Soil.

[58] Reetz H.F. (2017). Fertilizantes e o Seu uso Eficiente.

[59] Gautam A., Guzman J., Kovacs P., Kumar S. (2021). Manure and inorganic fertilization impacts on soil nutrients, aggregate stability, and organic carbon and nitrogen in different aggregate fractions. Arch. Agron. Soil Sci..

[60] Carmo D.L.D., De Lima L.B., Silva C.A. (2016). Soil Fertility and Electrical Conductivity Affected by Organic Waste Rates and Nutrient Inputs. Rev. Bras. De Ciência Do Solo.

[61] Nemali K. (2018). Details of Electrical Conductivity Measurements in Greenhouse Production.

[62] Haynes R., Naidu R. (1998). Influence of lime, fertilizer and manure applications on soil organic matter content and soil physical conditions: A review. Nutr. Cycl. Agroecosyst..

[63] Gross A., Glaser B. (2021). Meta-analysis on how manure application changes soil organic carbon storage. Sci. Rep..

[64] Corsi S., Friedrich T., Kassam A., Pisante M., Sà J.D.M. (2012). Soil Organic Carbon Accumulation and Greenhouse Gas Emission Reductions from Conservation Agriculture: A Literature Review.

[65] Luce M.S., Ziadi N., Chantigny M.H., Braun J. (2021). Long-term effects of tillage and nitrogen fertilization on soil C and N fractions in a corn–soybean rotation. Can. J. Soil Sci..

[66] Abagandura G.O., Mahal N.K., Butail N.P., Dhaliwal J.K., Gautam A., Bawa A., Kovács P., Kumar S. (2022). Soil labile carbon and nitrogen fractions after eleven years of manure and mineral fertilizer applications. Arch. Agron. Soil Sci..

[67] Whitney T. (2018). Building Soil Organic Matter Takes Time.

[68] Gonzatto R., Miola E.C.C., Doneda A., Pujol S.B., Aita C., Giacomini S.J. (2013). Volatilização de amônia e emissão de óxido nitroso após aplicação de dejetos líquidos de suínos em solo cultivado com milho. Ciência Rural.

[69] Sáez-Plaza P., Navas M.J., Wybraniec S., Michałowski T., Asuero A.G. (2013). An Overview of the Kjeldahl Method of Nitrogen Determination. Part II. Sample Preparation, Working Scale, Instrumental Finish, and Quality Control. Crit. Rev. Anal. Chem..

[70] Fangueiro D., Surgy S., Fraga I., Cabral F., Coutinho J. (2015). Band application of treated cattle slurry as an alternative to slurry injection: Implications for gaseous emissions, soil quality, and plant growth. Agric. Ecosyst. Environ..

